# Development of an indirect competitive chemiluminescent enzyme immunoassay for ethanamizuril residue detection in eggs and feed

**DOI:** 10.1016/j.fochx.2026.103838

**Published:** 2026-04-06

**Authors:** Xingdong Yang, Lihua Wu, Chenchen Wang, Yinuo Zhu, Yutong Cao, Keshi Ma, Lili Li, Xiaofei Hu

**Affiliations:** a College of Life Sciences and Agronomy, Zhoukou Normal University, Henan, Zhoukou 466001, China; b Key Laboratory of Animal Immunology of the Ministry of Agriculture, Henan Academy of Agricultural Sciences, Henan, Zhengzhou 450002, China

**Keywords:** Ethanamizuril, Monoclonal antibody, Chemiluminescent enzyme immunoassay (CLEIA), Quantitative detection, Feed, Eggs

## Abstract

Ethanamizuril (EZL), a newly developed antiparasitic drug, is expected to be widely used in poultry production in the future, but its residues in eggs and feeds pose potential risks to food safety. This study developed an indirect competitive chemiluminescent enzyme immunoassay (ic-CLEIA) for the quantitative detection of EZL residues in eggs and feeds. By optimizing the reaction conditions, the ic-CLEIA achieved a sensitivity (half-maximal inhibitory concentration, IC_50_) of 0.12 ng/mL and a linear range (IC_20_–IC_80_) of 0.02–0.92 ng/mL. Both the intra-assay and inter-assay coefficients of variation were less than 12%, demonstrating excellent reproducibility and high agreement with HPLC–MS/MS results. The developed ic-CLEIA filling the gap in rapid trace detection of EZL residues, provides a reliable technical support for food safety supervision of EZL residues, and expands the application of CLEIA in veterinary drug residue detection.

## Highlights

• A high-titre and sensitive monoclonal antibody against ethanamizuril (EZL) was developed.
• The sensitivity of the ic-CLEIA to detect EZL was 0.12 ng/mL.
• Target EZL in eggs and feed was successfully detected and verified.

## 1 Introduction

Chicken coccidiosis is a parasitic protozoan disease caused by *Eimeria coccidia* that parasitize the caecum of chickens. It is characterized by a wide range of prevalence, difficulty in prevention and control, and high mortality (Kim et al., 2021). It is difficult to provide comprehensive protection against coccidiosis using vaccines, and the host is still at risk of infection with coccidia (Zaheer et al., 2022). Therefore, anticoccidial drugs are still a reliable means of preventing and controlling coccidiosis in livestock and poultry. There are two main triazine anticoccidial drugs currently used in production: diclazuril and toltrazuril. However, owing to the excessive use of anticoccidial drugs in poultry farming, current clinical anticoccidial drug resistance is common, and the ecological environment is potentially poisoned (H. Zhang et al., 2023; Sun et al., 2023). New, safe and efficient anticoccidial drugs are urgently needed.

Ethanamizuril (EZL) is a new triazine anticoccidial drug independently developed by the Shanghai Veterinary Research Institute of the Chinese Academy of Agricultural Sciences and is based on diclazuril and toltrazuril (Chen et al., 2024). The drug has a unique chemical structure. Preliminary experiments revealed that a dose of 5 mg/kg added to feed or drinking water has a good anticoccidial effect. A dose of 9 mg/kg or more added to feed or drinking water can stably achieve a high-efficiency anticoccidial effect, with an anticoccidial index (ACI) of 185–200 (Zhang et al., 2019). Studies have confirmed that it has the characteristics of low dosage and high anticoccidial activity and was officially approved for production by the Ministry of Agriculture and Rural Affairs of China in March 2022. As of February 2026, EZL has not had any publicly available records of full approval for marketing or clear official regulatory registration outside China. In regions such as the European Union, the United States, Japan, and Australia, it remains in a state of having no explicit regulatory approval or formal inclusion in the local regulatory framework. Triazine anticoccidial drugs can inhibit the activity of respiratory chain enzymes of coccidia and inhibit oxidative phosphorylation in the mitochondria of coccidia cells, leading to the obstruction of coccidia respiration, thereby affecting the normal metabolism of the parasite, causing death (Li et al., 2019; Li et al., 2020).

Humans may be continuously exposed to EZL through long-term consumption of poultry meat or eggs containing trace amounts of EZL residues. At present, research data on the specific effects of such long-term, low-dose exposure on human health (such as carcinogenicity, reproductive toxicity, developmental toxicity, and endocrine disruption) are insufficient (Zhang, Wang, Li, et al., 2020; Zhang, Wang, Wang, et al., 2020). Some animal experimental studies have shown that EZL and its metabolites may have certain thyroid-disrupting effects (Sinkó et al., 2022). In addition, the widespread use of EZL may promote the emergence of drug-resistant parasites and even drug-resistant bacteria (Cheng, Wang, et al., 2022; Xiao et al., 2019). China prohibits the use of EZL during the laying period. The maximum residue limits (MRLs) of EZL in chicken muscle, sebum, liver, and kidney are 100, 300, 200, and 600 μg/kg, respectively. However, with the development of the poultry industry, the use of EZL will become more widespread. Therefore, it is crucial to develop sensitive and accurate detection methods to regulate EZL residues in poultry products.

Currently, methods for detecting EZL residues include ultrahigh-performance liquid chromatography (UPLC)–ultraviolet (UV) detection (Zhao et al., 2017) and UPLC–mass spectrometry (Cheng, Zheng, et al., 2022; Li et al., 2022; Wang et al., 2019). These methods are highly sensitive and provide accurate and reliable test results, but they require high pretreatment and strong professional operation and cannot achieve high-throughput on-site screening. Chemiluminescence enzyme immunoassays (CLEIAs) have the advantages of being low cost and high throughput and complement the advantages of large-scale instrument confirmation methods, significantly improving the detection efficiency of veterinary drug residues in livestock, poultry products, and feed. As a newly developed triazine antiparasitic drug, the current research on EZL has only focused on the analysis of its antiparasitic activity and mechanism of action. Although CLEIA detection technology has been widely used in clinical medicine, food safety, environmental monitoring, and other fields for rapid trace detection (Guo et al., 2023; Kyme et al., 2019; Thongpradit et al., 2022), systematic database searches have confirmed that no reports on the establishment, validation and application of CLEIA detection methods for EZL are currently available.

In this study, highly specific monoclonal antibodies against EZL were screened and prepared, and an indirect competitive CLEIA (ic-CLEIA) for detecting EZL content in eggs and laying hen feed was performed to provide technical support for food safety supervision.

## 2 Materials and methods

### 2.1 Materials and apparatus

EZL [(CAS 1560840–75-6, molecular weight (MW): 352.34 g/mol)] was provided by the CATO (Guangzhou, Guangdong, China). Decoquinate (CAS 18507–89-6, MW: 417.54 g/mol, CID: 29112), metamitron (CAS 41394–05-2, MW: 202.21 g/mol, CID: 38854), diclazuril (CAS 101831–37-2, MW: 407.64 g/mol, CID: 456389), and toltrazuril (CAS 69004–03-1, MW: 425.38 g/mol, CID: 68591) were purchased from Macklin (Shanghai, China); metronidazole (CAS 443–48-1, MW: 171.15 g/mol, CID: 4173), N-hydroxysuccinimide (NHS) (CAS 6066-82-6, MW: 115.09 g/mol, CID: 80170), and actarit (CAS 18699–02-0, MW: 193.20 g/mol, CID: 2018) were obtained from Aladdin (Shanghai, China). Freund's complete adjuvant (FCA), goat ant-mouse IgG-horseradish peroxidase (HRP), 1-ethyl-3-(3-dimethylaminopropyl) carbodiimide (EDC) (CAS 25952–53-8, MW: 191.70 g/mol, CID: 15908) and Freund's incomplete adjuvant (FIA) were purchased from Sigma–Aldrich (St. Louis, MO, U.S.); low molecular weight protein marker, microplate (96 wells), 3,3,5,5-tetramethylbenzidine (TMB) two-component substrate solution for ELISA, and Ultra-Sensitive Chemiluminescent Substrate were obtained from Solarbio (Beijing, China). White light-emitting panels were obtained from Jingan (Shanghai, China); and bovine serum albumin (BSA) (CAS 9048-46-8, MW: 66446 g/mol) and ovalbumin (OVA) (CAS 9006-59-1, MW: 44500 g/mol) were obtained from Yuanye (Shanghai, China). RPMI-1640, HT, and HAT culture media were purchased from Proteintech (Wuhan, China); *N*, *N*-dimethylformamide (CAS 68–12-2, MW: 73.09 g/mol, CID: 6228), TMB (CAS 54827–17-7, MW: 240.34 g/mol, CID: 66649627), and PEG1500 were purchased from Sinopharm (shanghai, China). Eight-week-old female BALB/c mice were obtained from the Laboratory Animal Center of Zhengzhou University (Zhengzhou, Henan, China) and were raised according to principles of the Biomedical Ethics Committee of Zhoukou Normal University (ZKNU-2024086).

A Multiskan FC microplate reader was obtained from Thermo Fisher Scientific (Shanghai, China). An ES-E220B/ES-E320B electronic analytical balance was purchased from Deante (Tianjin, China). A SpectraMaxi3x multifunctional microplate reader was purchased from Molecular Devices (Sunnyvale, CA, USA). The manual pipette was purchased from Eppendorf (Hamburg, Germany). A UPLC H-Class-TQD MS system was purchased from Waters Corporation (Milford, MA, USA).

### 2.2 Modification of the hapten EZL

**Fig. 1:**
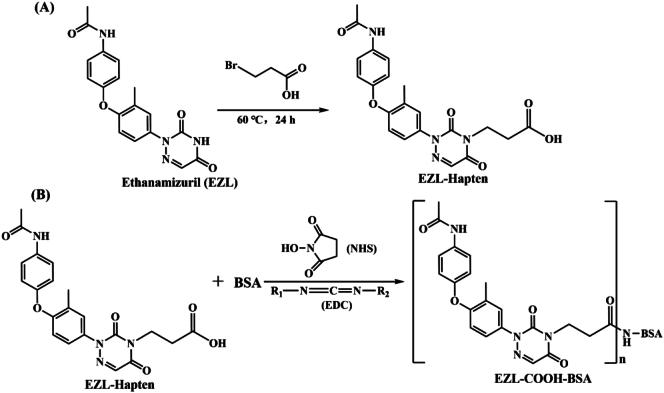
(A) The hapten ethanamizuril (EZL) was modified to introduce a carboxyl group. (B) Preparation of the complete antigen of ethanamizuril (EZL-BSA) by the active ester method.

First,177 mg of EZL, 85 mg of 3-bromopropionic acid, and 152 mg of potassium carbonate were dissolved in 2 mL of *N*, *N*-dimethylformamide and refluxed at 60 °C for one day. After the reaction was complete, the mixture was cooled to room temperature, the pH was adjusted to approximately 1.5 with 1 mol/L hydrochloric acid, and the solution was extracted with 15 mL of ethyl acetate three times. The organic phase was washed three times with 15 mL of sodium chloride aqueous solution and dried over anhydrous sodium sulfate. The final solution was concentrated in vacuo to obtain the EZL hapten (Fig. 1A).

### 2.3 Synthesis of EZL immunogen and antigen detection

First, 35.3 mg of EZL was dissolved in 3.5 mL of *N*, *N*-dimethylformamide. Next, 18.6 mg of EDC and 17.3 mg of NHS were added, and the mixture was shaken and reacted at room temperature in the dark for 4 h to obtain activation solution A. Sixty-six milligrams of BSA was dissolved in a phosphate-buffered saline (PBS, pH 7.4) solution to obtain solution B. Under an ice bath, activation solution A was slowly added to solution B, and the mixture was allowed to react at room temperature in the dark for 4 h to obtain solution C. Solution C was dialyzed with a dialysis bag in dialysate (1000 mL PBS, 4 °C) for 3 days, filtered and sterilized, and stored in a − 20 °C refrigerator (Fig. 1B) (Wang et al., 2020). The above steps were repeated, and the BSA was replaced with OVA to obtain the detection antigen of EZL (EZL-OVA). The synthesized antigen was identified by UV spectral scanning. The identification method was as follows: PBS was used to prepare BSA, OVA, EZL, EZL-BSA, and EZL-OVA solutions with a mass concentration of 1.0 mg/mL. After the baseline was zeroed, the above five solutions were scanned by UV spectroscopy in the wavelength range of 220–350 nm, and the absorbance at different wavelengths was recorded. The data were analysed, and the UV spectra were combined. The combined graph was used to analyse whether EZL was coupled with BSA and OVA.

### 2.4 Animal immunization and preparation and characterization of monoclonal antibodies against EZL

Three 8-week-old female BALB/c mice (#1–3) were immunized with the prepared EZL-BSA complete antigen. The immunization methods included a low dose (78 μg/mouse), a long interval (3 weeks), multiple subcutaneous injections at the back of the mice (4–6 points), and multiple injections (5 times). After all immunization procedures were completed, the immunized mice were tail cut, and blood was collected to identify anti-EZL polyclonal antibodies (pAbs). Immunized mice with high titres and low half maximum inhibitory concentrations (IC_50_s) were selected as cell fusion mice. Mice were euthanized with isoflurane anesthesia for three minutes, followed by cervical dislocation. Then splenectomy was performed. Cell fusion was performed according to conventional methods (Luo et al., 2024). Before fusion, NS0 cells were resuscitated, mouse spleens were removed under sterile conditions, and spleen cells were isolated and fused with NS0 cells under the action of PEG-1500. Cell lines with high titres and good sensitivity were selected for expansion culture, and anti-EZL monoclonal antibodies (mAbs) were prepared by the in vivo ascites induction method. The collected ascites was purified by the saturated octanoic acid–ammonium sulfate method. The titres of anti-EZL mAb in the cell culture supernatant and ascites were determined by indirect enzyme-linked immunosorbent assay (ELISA). Sensitivity was determined by ic-ELISA (Tomiki et al., 2024) to determine the IC_50_ value of the anti-EZL mAb against EZL.

### 2.5 Ic-CLEIA operation steps for EZL detection

The operation steps of the ic-CLEIA were as follows: Step 1 (coating): the detection antigen (EZL-OVA) was diluted to a concentration of 5.0 μg/mL with 0.05 mol/L carbonate buffer (CBS, pH 9.6) and coated on the chemiluminescent enzyme-labelled plate at 65.0 μL/well. The plate was incubated at 37.5 °C for 2 h, followed by washing 4 times with 0.01 mol/L phosphate buffer containing 0.05% Tween-20 (PBST, pH 7.4). Step 2 (blocking): blocking solution (PBS containing 5% skim milk powder) was added at 225.0 μL/well and incubated at 37.5 °C for 1 h. Step 3: Different concentrations of EZL and anti-EZL mAb were added to each well and incubated at 65.0 μL/well at 37.5 °C for 20 min, followed by washing 4 times with PBST. Step 4: sixty-five microlitre of 2000-fold diluted goat anti-mouse IgG-HRP (GaM-IgG-HRP) was added to each well, and the samples were incubated at 37.5 °C for 30 min and then washed 6 times with PBST. Step 5: sixty-five microlitre/well of ultra-sensitive chemiluminescent solution was added to the chemiluminescent ELISA plate and allowed to stand at room temperature for 6 min. Step 6: the relative light unit (RLU) value was obtained using a multimode microplate reader, and the results were determined.

### 2.6 Establishment of optimal ic-CLEIA reaction conditions

The mass concentrations of the coated antigen (1.25, 2.50, 5.0, and 7.5 μg/mL) and the antibody dilution (1:1000, 1:2000, 1:4000, 1:8000, and 1:16000) were selected using the chessboard method, and the optimal reaction conditions were determined on the basis of the maximum ratio (P/N) of the positive control RLU value (P) and the negative control RLU value (N).

Single-factor experiments were performed to optimize the influencing factors, such as coating conditions (37 °C for 1 h, 37 °C for 2 h, and 4 °C for 9 h), blocking solution (5% skim milk, 1% gelatine, and 1% casein), blocking conditions (37 °C for 0.5 h, 37 °C for 1 h, and 37 °C for 1.5 h), primary antibody competition reaction time (10, 20, and 30 min), GaM-IgG-HRP dilution (1000, 2000, 4000, and 8000 times), and substrate color development time (4, 6, and 8 min). The optimal reaction conditions should have an appropriate absorbance value (RLUmax), a lower IC_50_, and a higher RLUmax/IC_50_ ratio. The higher the RLUmax/IC_50_ is, the lower the IC_50_; that is, the higher the sensitivity.

### 2.7 Pretreatment of the sample

Fresh egg and basic feed blank matrix models were established to evaluate the per-formance of the CLEIA for EZL residue detection in eggs and feed. Sixty commercial eggs were randomly sampled from three local supermarkets in Chuanhui District, Zhoukou, Henan Province, China, and confirmed to be free of EZL residues by HPLC-MS/MS. The whole shelled egg was homogenized with a homogenizer at high speed to form a uniform slurry. A total of 4.0 g of the homogenized sample was placed in a 50 mL centrifuge tube. Then, 24.0 mL of acetonitrile-double distilled water (80:20, *v*/v) was added, and the mixture was vortexed for 2 min and centrifuged at 4500 r/min for 5 min at 4 °C. Then, 12.0 mL of n-hexane was added to the supernatant and vortexed for 5 min. The supernatant was allowed to stand for stratification, and the upper n-hexane layer was discarded. The degreasing was repeated once. Four millilitres of the lower layer of extract was removed from the nitrogen blowpipe and blown to near dryness at 40 °C. The residue was redissolved in 1 mL of PBS and vortexed for 1 min. The sample was filtered through a 0.22 μm water filter membrane and tested.

Blank laying hen feed (2.0 g) was placed in a 50 mL stoppered centrifuge tube, 10 mL of acetonitrile was added, the mixture was vortexed for 1 min, and the mixture was centrifuged at 4000 r/min for 10 min; 5 mL of the supernatant acetonitrile layer was placed in a small test tube and dried with nitrogen in a 50 °C water bath. After redissolving with 1 mL of an acetonitrile–0.2% phosphoric acid water mixture (*V*/V, 40:60), 1 mL of n-hexane was added, the mixture was vortexed for 1 min, the upper n-hexane layer was discarded, and the lower layer of liquid was removed and centrifuged at 6000 r/min for 10 min and filtered through a 0.22 μm microporous filter membrane for testing.

### 2.8 Performance evaluation of the EZL–ic-CLEIA

The ic-ELISA standard curve of EZL was established on the basis of the optimal reaction conditions, where the vertical coordinates B and B0 represent the RLU values when EZL was added and not added, respectively, and the horizontal coordinate represents the mass concentration of EZL (0, 5.0, 10.0, 20.0, 40.0, 80.0, 160.0, 320.0, 640.0, 1280.0, 2560.0, and 5120.0 pg/mL, respectively).

The accuracy and precision were determined by the addition recovery test. EZL was added to the blank samples of laying hen feed and eggs, and three concentrations, namely, low, medium, and high (*n* = 6 per level), and six replicates were established for each batch. In the process of laboratory model construction, random sampling was adopted for the collection of eggs and feed treated with EZL. Ic-CLEIA was used for detection, and the addition recovery rate and coefficient of variation (CV) were calculated.

Specificity was determined by using a cross-reactivity (CR) test to determine the cross-reactivity rate (CRR) of the EZL mAb to EZL and other compounds to evaluate the specificity of the EZL mAb and calculate the CRR. CRR (%) = IC_50_ EZL (ng/mL)/IC_50_ other analogues (ng/mL) × 100%.

The reliability of the assay was determined by comparing the EZL–ic-ELISA and HPLC–MS/MS analytical methods. Twelve samples of laying hen feed and eggs were purchased locally. All the samples were divided into two groups, one of which was tested by EZL–ic-ELISA and the other by HPLC–MS/MS, and the test results were compared.

### 2.9 Data analysis

The experimental data were analysed and processed using Excel 2021 and are expressed as the mean ± standard deviation; ChemDraw 2022 and GraphPad Prism 8 software were used for graphing.

## 3 Results and discussion

### 3.1 Identification of coupled antigens and polyclonal antiserum

**Fig. 2:**
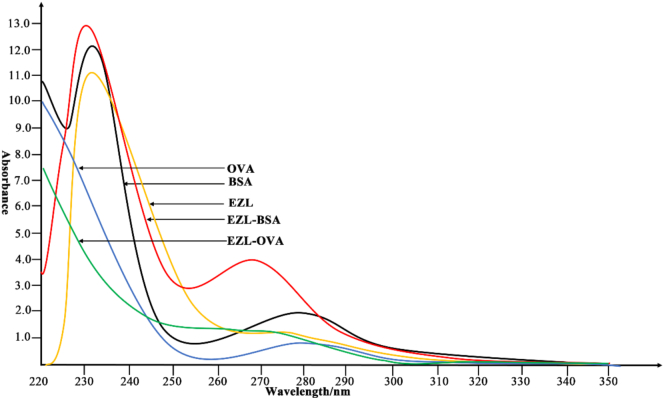
Ultraviolet scanning spectra of ethanamizuril (EZL), bovine serum albumin (BSA), EZL-BSA, ovalbumin (OVA), and EZL-OVA.

**Fig. 3:**
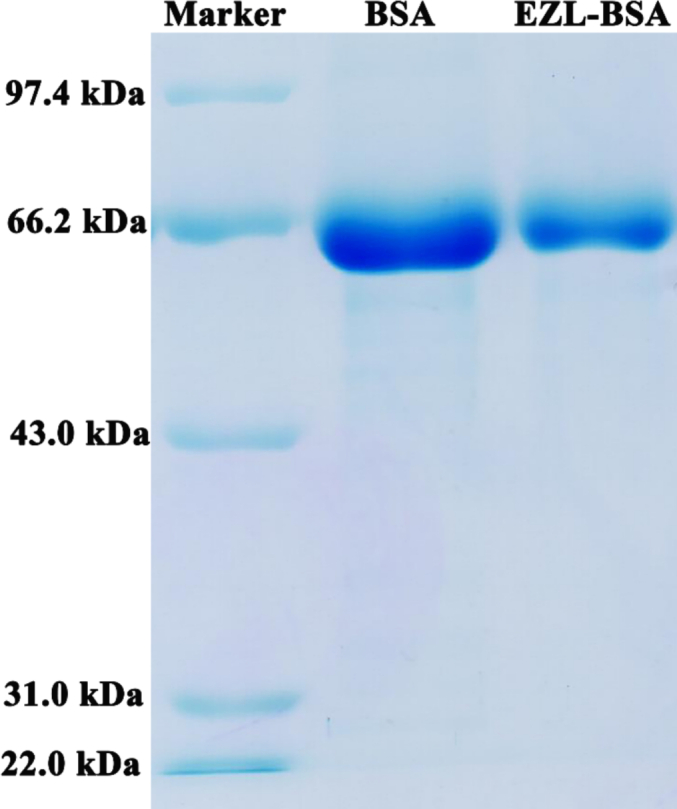
Sodium dodecyl sulfonate–polyacrylamide gel electrophoresis patterns of ethanamizuril (EZL), bovine serum albumin (BSA), and EZL-BSA.

The molecular weight of EZL is 352.34, and possesses only reactogenicity. If animals are directly immunized with EZL, no antibodies can be produced. However, EZL cannot be directly coupled to the carrier protein; thus, we modified EZL and introduced a carboxyl group. When EZL is coupled with a carrier protein (>10 kDa), pAbs against EZL can be produced after the immunization of animals, which provides the possibility for the subsequent preparation of mAbs against EZL. The UV scanning spectrum (Fig. 2) revealed that EZL had a characteristic absorption at 230 nm, and the absorption peaks of EZL-BSA and EZL-OVA were red-shifted relative to the absorption peak of BSA (280 nm) and OVA (278 nm), indicating that EZL and the carrier protein were successfully coupled. The results of sodium dodecyl sulfonate–polyacrylamide gel electrophoresis showed that the migration rate of the conjugate product of EZL hapten and carrier was slightly lower than that of the carrier protein, indicating that the molecular weight of the conjugate was larger than that of BSA, further proving that the artificial antigen of EZL was successfully prepared (Fig. 3).

After the immunization procedure, the titre and sensitivity (IC_50_) of the mouse antiserum were tested. The results revealed that the titre/IC_50_ of mouse antiserum in three parallel experiments was 5.12 × 10^4^/13.89 ng/mL, 2.56 × 10^4^/32.72 ng/mL and 2.56 × 10^4^/44.31 ng/mL, respectively (Table 1). The higher the titre and the smaller the IC_50_, the better the affinity and sensitivity of the antiserum to recognize EZL.

**Table 1:** Titre and sensitivity of anti-ethanamizuril polyclonal antiserum from immunized mice No. 1–3.

dilution multiple	Titre of polyclonal antiserum		
	No.1	No.2	No.3
2.0 × 10^2^	3.643	2.779	2.749
4.0 × 10^2^	3.315	2.441	2. 310
8.0 × 10^2^	2.958	2.121	2.043
1.6 × 10^3^	2.426	1.776	1.626
3.2 × 10^3^	1.898	1.402	1.390
6.4 × 10^3^	1.411	1.059	0.986
1.28 × 10^4^	0.996	0.698	0.650
2.56 × 10^4^	0.556	0.334	0.329
5.12 × 10^4^	0.267	0.165	0.133
Positive control	0.097	0.088	0.081
Blank control	0.072	0.076	0.063
IC_50_ (ng/mL)	13.89	32.72	44.31

### 3.2 Preparation of monoclonal antibodies against EZL

The properties of the antibody directly determine the sensitivity and specificity of the method and are crucial for the performance of the assay. Mice with a titre/IC_50_ of 5.12 × 104/3.89 ng/mL were selected for cell fusion. Positive cell wells and resistant cell wells were screened by ic-ELISA. Three hybridoma cell fusion wells showed strong positivity and EZL resistance. The limiting dilution method was used for multiple cloning and screening. The titre determined by indirect ELISA was used as the screening standard to select hybridoma cell lines with higher titres. After multiple screenings, three monoclonal hybridoma cell lines (1F7, 2D2, and 3E5) with higher titres and stronger specificity were obtained. Only hybridoma clones that satisfied all the above criteria–high reactivity with the immunizing conjugate, no non-specific binding, and good competitive inhibition by the target analyte–were judged as specific mAbs-producing clones. The titres of the cell culture supernatant and ascites were distributed in the ranges of 1:4.0 × 10^2^–1:1.6 × 10^3^ and 1:1.28 × 10^5^–1:5.12 × 10^5^, respectively. Among them, the cell line with the strongest specificity was 2D2. After the culture was expanded, the anti-EZL mAbs were prepared by the ascites method. The IC_50_ values of the three types of ascites were 9.74, 3.23, and 12.44 ng/mL, respectively.

### 3.3 Optimization of the working conditions of EZL–ic-CLEIA

In chemiluminescent enzyme immunoassay, the optimization of coating antigen concentration and antibody dilution should be systematically screened by chessboard titration to achieve a superior IC_50_ value, minimal background signal, and the widest linear detection range, thereby ensuring the sensitivity, specificity, and quantitative accuracy of the method. According to the maximum P/N value, the best reaction conditions were screened by chessboard titration; thus, the coating concentration of the detection antigen (EZL-OVA) was determined to be 5.0 μg/mL, and the anti-EZL mAb was diluted 8000 times (Table 2).

**Table 2:** Checkerboard titration data of the indirect competitive chemiluminescent enzyme immunoassay.

Concentration of coating antigen (μg/mL)	Anti-ethanamizuril monoclonal antibody dilution multiple									
	1000		2000		4000		8000		16,000	
	P	P/N	P	P/N	P	P/N	P	P/N	P	P/N
1.25	901,254	25.27	849,648	28.66	691,291	32.64	464,076	30.88	276,226	30.09
2.5	920,587	19.23	866,939	25.36	707,440	24.44	552,102	31.76	326,332	31.28
5.0	956,145	17.07	876,568	20.52	738,789	23.07	676,568	35.43	393,028	24.82
7.5	979,792	15.10	893,135	17.16	778,664	18.42	686,095	27.06	420,291	19.66

**Fig. 4:**
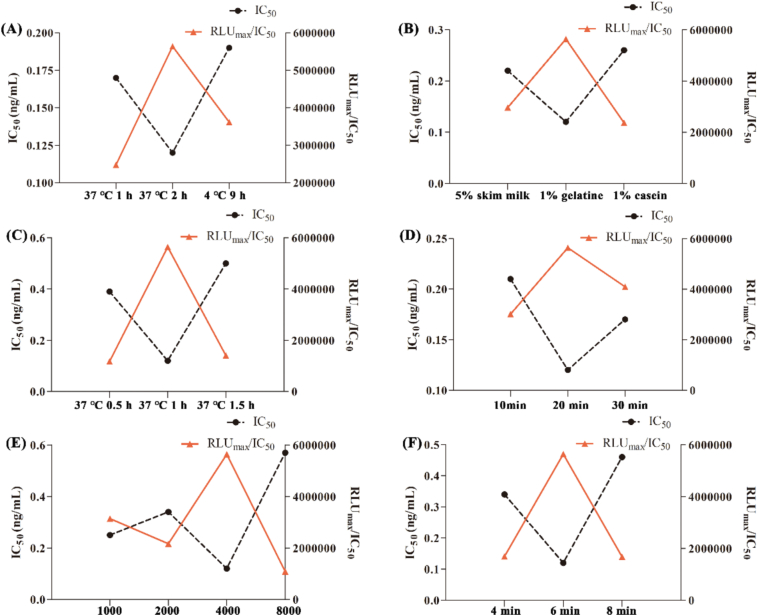
Optimization of working conditions for indirect competitive chemiluminescence enzyme immunoassay. (a) Coating conditions for ethanamizuril-ovalbumin (EZL-OVA). (b) Selection of blocking solution. (c) Optimization of the closure conditions of the white luminescence ELISA plate. (d) Optimization of the competition time between the anti-EZL monoclonal antibody and EZL. (e) Optimization of the dilution of goat anti-mouse horseradish peroxidase-immunoglobulin G. (f) Effect time of the chemiluminescent substrate.

The optimal experimental conditions for the single-factor experiments were determined by the higher RLU_max_/IC_50_ and the lower IC_50_ values. Compared with incubation at 37 °C for 1 h, coating at 37 °C for 2 h resulted in more sufficient immunoreaction and a higher RLU_max_/IC_50_ value, while it took less time and was more convenient to operate than 4 °C for 9 h (Fig. 4A); the white luminescent plate was blocked with 1% gelatine at 37 °C for 1 h (Fig. 4B, C). Gelatine as a blocking solution exhibits lower background, less non-specific adsorption, better signal stability and less interference with immunoreactions compared with skimmed milk powder and casein. After comprehensive comparison of different blocking durations, blocking at 37 °C for 1 h not only achieves more sufficient blocking and effectively reduces non-specific adsorption compared with 0.5 h, but also avoids elevated background and increased experimental time caused by excessively long blocking for 1.5 h, thereby achieving an optimal balance between blocking effect and assay efficiency; A reaction time of 20 min between the anti-EZL mAb and EZL was selected, which allowed more sufficient and stable reaction than 10 min, while avoiding elevated background and prolonged assay time associated with a 30-min reaction (Fig. 4D); A 4000-fold dilution of GaM-IgG-HRP was selected, which provided lower background and less non-specific binding than 1000-fold and 2000-fold dilutions, as well as stronger signal and better sensitivity than 8000-fold dilution, showing the best overall performance (Fig. 4E); Six minutes was selected as the optimal color development time of the substrate, which could achieve higher signal intensity and better stability compared with 4 min, while avoiding signal saturation and elevated background caused by prolonged incubation for 8 min (Fig. 4F).

### 3.4 Performance test results of the EZL-ic-CLEIA

**Fig. 5:**
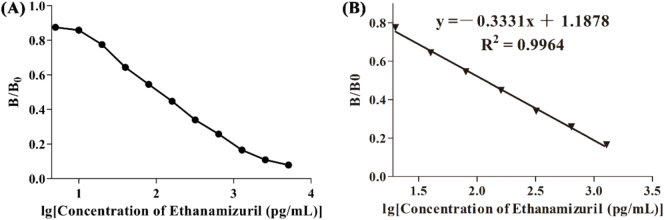
(A) The standard curve of ethanamizuril (EZL) was generated by indirect competitive chemiluminescence enzyme immunoassay (ic-CLEIA). (B) Calibration curve of EZL detected by ic-CLEIA.

Under the optimal conditions of the above working system, the standard curve (Fig. 5A) and calibration curve (Fig. 5B) of EZL-ic-CLEIA were established, and its IC_50_ for EZL was 0.12 ng/mL; the linear detection range (IC_20_–IC_80_) was 0.02–0.92 ng/mL, and the detection limit (IC_10_, LOD) was 0.007 ng/mL.

The established ic-CLEIA method was used to perform six repetitive tests on two EZL samples of different concentrations. The average recovery of three solution samples with different concentrations was calculated, and the intra-assay and inter- assay CV values were analysed. The average intra-assay and inter-assay addition recoveries were 80.6%–91.9% and 80.0%–90.5%, respectively, and the CVs were 5.5–9.8% and 6.3–11.8%, respectively. The average recoveries of the eggs in the intra-assay and inter-assay groups were 81.9%–94.9% and 79.9%–93.4%, respectively, both of which were within the normal range; the CVs were 4.9%–9.1% and 5.5–10.2%, respectively, both of which were less than 12% (Table 3). The above preliminary results reveal that the accuracy and precision of the established method are good.

**Table 3:** Indirect competitive chemiluminescent enzyme immunoassay for the detection of ethanamizuril in feed and eggs using a recovery test.

samples	Spiked BAC (ng/mL)	Intraassay			Interassay		
		Mean ± SD (ng/mL)	Recovery (%)	CV (%)	Mean ± SD (ng/mL)	Recovery (%)	CV (%)
Feed	0.24	0.19 ± 0.02	80.6 ± 7.9	9.8	0.19 ± 0.02	80.0 ± 9.6	11.8
	12	10.17 ± 0.83	84.8 ± 6.9	8.2	10.03 ± 0.87	83.6 ± 8.3	8.7
	48	44.09 ± 2.41	91.9 ± 5.0	5.5	43.42 ± 2.74	90.5 ± 5.7	6.3
eggs	0.24	0.20 ± 0.02	81.9 ± 7.5	9.1	0.19 ± 0.02	79.9 ± 8.1	10.2
	12	10.32 ± 0.72	86.0 ± 6.0	7.0	10.20 ± 0.75	85.0 ± 6.3	7.4
	48	45.53 ± 2.25	94.9 ± 4.7	4.9	44.83 ± 2.47	93.4 ± 5.1	5.5

The specificity of the antibody can be evaluated by measuring the cross-reaction rate (CR) of the antibody to the analogue. The lower the CR is, the greater the specificity of the antibody to the target drug. The experimental results show that with EZL as the reference, the CR of the established ic-CLEIA method with EZL was 100%, and there was essentially no cross-reaction with other structural and functional analogues, such as decoquinate, metamitron, diclazuril, toltrazuril, metronidazole, and actarit (Table 4), indicating that the established ic-CLEIA method has good specificity for EZL.

**Table 4:** Determination of the cross-reaction of ethanamizuril structural analogues based on an indirect competitive chemiluminescent enzyme immunoassay.

Compounds tested	structural analogues	IC_50_ (ng/mL)	Cross-reactivity (%)
Ethanamizuril		0.1	100
diclazuril		>1.0 × 10^3^	<0.01
metamitron		>1.0 × 10^3^	<0.01
toltrazuril			
decoquinate		>1.0 × 10^3^	<0.01
metronidazole		>1.0 × 10^3^	<0.01
actarit		>1.0 × 10^3^	<0.01

Two negative blank samples, laying hen feed and eggs, were selected, and high, medium, and low concentrations were added. After the two samples were treated, their recoveries were detected by the ic-CLEIA method and the HPLC–MS/MS method. The results revealed that the recoveries of the ic-CLEIA method in feed and eggs were 83.1%– 93.9% and 84.3%–94.9%, respectively, and the CV was <10%, which was essentially consistent with the HPLC–MS/MS results (Table 5). These findings revealed that the results of the established ic-CLEIA method were accurate and reliable.

**Table 5:** Comparison between indirect competitive chemiluminescent enzyme immunoassay (ic-CLEIA) and HPLC–MS/MS.

samples	Spiked BAC (ng/mL)	ic-CLEIA			HPLC		
		Mean ± SD (ng/mL)	Recovery (%)	CV (%)	(ng/mL)	Recovery (%)	CV (%)
feed	10	8.31 ± 0.73^*a*^	83.1 ± 7.3	8.8	8.67 ± 0.62 ^*a*^	86.7 ± 6.2	7.2
	30	27.23 ± 1.92 ^*a*^	90.8 ± 6.4	7.1	28.69 ± 1.81 ^*a*^	95.6 ± 6.0	6.3
	90	84.48 ± 4.10 ^*a*^	93.9 ± 4.6	4.9	86.99 ± 3.46 ^*a*^	96.7 ± 3.8	4.0
eggs	10	8.43 ± 0.64 ^*a*^	84.3 ± 6.4	7.6	8.74 ± 0.52 ^*a*^	87.4 ± 5.2	5.9
	30	27.38 ± 1.59 ^*a*^	91.3 ± 5.3	5.8	28.79 ± 1.16 ^*a*^	96.0 ± 3.9	4.0
	90	85.41 ± 3.60 ^*a*^	94.9 ± 4.0	4.2	87.67 ± 2.71 ^*a*^	97.4 ± 3.0	3.1

*a* in the upper right indicates no statistically significant difference between ic-CLEIA and HPLC-MS/MS (*P* > 0.05).

## 4 Conclusion

The ic-CLEIA developed in this study had an IC_50_ of 0.12 ng/mL, a linear detection range of 0.02–0.92 ng/mL (IC_20_–IC_80_), and a limit of detection of 0.007 ng/mL, demonstrating high sensitivity for EZL detection. The recoveries in the feed and eggs ranged from 80.6% to 91.9% and from 81.9% to 94.9%, respectively, demonstrating the high accuracy of the assay. Precision tests revealed intra-assay and inter-assay CVs below 12%, indicating good precision for the detection of EZL. The cross-reactivity with structurally similar analogues of EZL was less than 0.1%, confirming the high specificity of the assay. The CLEIA established in this study provides an efficient detection method for EZL residues in eggs and feeds. This enables domestic regulatory authorities to conduct large-scale sample screening, identify non-compliant products in a timely manner, and strengthen the food safety barrier in China. Meanwhile, it offers technical reference for the detection of other anticoccidial drugs and veterinary drug residues.

In future studies, the development of chemiluminescent enzyme immunoassay will focus on the following aspects: (1) Screening and developing novel labelling enzymes and substrates with high quantum yield, and optimizing the reaction system to further improve detection sensitivity and signal stability; (2) Developing miniaturized and portable detection devices to achieve point-of-care testing for on-site rapid detection; (3) Exploring multiplex detection technologies to enable simultaneous determination of multiple targets in a single run, thereby reducing detection cost and improving analytical efficiency. However, we also recognize several key limitations of chemiluminescent enzyme immunoassays that need to be addressed in future research: (1) Enzyme activity is susceptible to interference from temperature, pH, and sample matrix effects, which may lead to distorted detection signals; (2) Reagents require strict storage conditions and rely heavily on dedicated instruments, increasing application costs and limiting popularization at the grassroots level; (3) The method may suffer from non-specific signal amplification and the hook effect, which affect detection accuracy and reliability.

## CRediT authorship contribution statement

**Xingdong Yang:** Writing – original draft, Visualization, Project administration, Methodology, Funding acquisition, Formal analysis, Conceptualization. **Lihua Wu:** Validation, Investigation, Data curation. **Chenchen Wang:** Validation, Investigation, Data curation. **Yinuo Zhu:** Investigation, Data curation. **Yutong Cao:** Investigation, Data curation. **Keshi Ma:** Supervision, Resources. **Lili Li:** Supervision, Resources. **Xiaofei Hu:** Writing – review & editing, Supervision, Resources, Project administration, Methodology, Funding acquisition, Conceptualization.

## Ethical approval

All international, national, and/or institutional guidelines applicable to procedures carried out in the present study were followed.

## Declaration of competing interest

The authors declare that they have no known competing financial interests or personal relationships that could have appeared to influence the work reported in this paper.

## Data Availability

Data will be made available on request.
