# Supplementary material for: Development of an indirect competitive chemiluminescent enzyme immunoassay for ethanamizuril residue detection in eggs and feed
Source: Food Chem X. 2026 Apr 6;35:103838. doi: 10.1016/j.fochx.2026.103838 (PMC13091162; doi:10.1016/j.fochx.2026.103838)
Supplement: Supplementary file 2 — Supplementary material 2 [file mmc2.zip › mmc2/Description of the Interactive data visualization File.docx]

**Interactive data visualization file**

Fig. S1 Standard curve of ethanamizuril by indirect competitive enzyme-linked immunosorbent assay (1#)

Fig. S2 Standard curve of ethanamizuril by indirect competitive enzyme-linked immunosorbent assay (2#)

Fig. S3 Standard curve of ethanamizuril by indirect competitive enzyme-linked immunosorbent assay (3#)

Fig. S4 Standard curve of ethanamizuril by indirect competitive enzyme-linked immunosorbent assay (1F7)

Fig. S5 Standard curve of ethanamizuril by indirect competitive enzyme-linked immunosorbent assay (2D2)

Fig. S6 Standard curve of ethanamizuril by indirect competitive enzyme-linked immunosorbent assay (3E5)

Fig. S7 Standard curve of ic-CLEIA for the detection of ethanamizuril

Fig. S8 Calibration curve of ic-CLEIA for the detection of ethanamizuril
